# Psychological autopsy studies of physician and nurse suicide: A narrative summary and synthesis of circumstantial and psychological aspects of death

**DOI:** 10.1177/22799036261465285

**Published:** 2026-07-03

**Authors:** Elizabeth Kreuze, Elizabeth I. Merwin, Maureen Underwood, Janet York

**Affiliations:** 1 College of Nursing and Health Innovation, 12329 University of Texas at Arlington, Arlington, TX, USA; 2 LLC Suicide Prevention Specialists, Underwood & Associates, Dallas, TX, USA; 3 College of Nursing, 2345 Medical University of South Carolina, Charleston, SC, USA

**Keywords:** psychological, autopsy, physician, nurse, occupation, suicide

## Abstract

The aim of this review was to identify and examine psychological autopsy studies of physician and nurse suicide, with the overriding goal of better understanding circumstantial and psychological aspects of death. To the best of our knowledge, this represents the first review to examine psychological autopsy studies of physicians and nurses. A variety of search strategies were utilized. A literature search was conducted using medical databases, reference lists from articles were hand-searched, articles from academic websites were hand-searched, and articles from organizational websites were hand-searched. In total, eight psychological autopsy studies on physician suicide and one psychological autopsy study on nurse suicide were included. Physician and nurse suicide decedents from England and Wales, Finland, Thailand, and the United States were examined using psychological autopsy. Sociodemographic characteristics, occupational status, method(s) of suicide, location of suicide, and presence of a suicide note were inconsistently reported. Alternatively, histories, contributing factors, and circumstances preceding death were consistently reported. Specifically, physician and nurse suicide decedents often had history of mental health concerns, psychiatric hospitalization, and suicide attempt(s). Physician and nurse suicide decedents also often experienced current mental health concerns at time of death, particularly depressive disorders. Alcohol and substance use disorder sometimes occurred concomitantly. Taken together, additional research may be important in updating the evidence base. Additional psychological autopsy studies that include physician and nurse suicide decedents from different global regions may also be important in building the evidence base and in determining the generalizability of findings. Collectively, additional research is important, as psychological autopsy studies may provide important implications for research, surveillance, prevention, and intervention.

## Introduction

International meta-analytic evidence suggests female and male physicians experience higher suicide mortality than the female and male general population, respectively.^1,2^ This global evidence further suggests risk of suicide among female physicians is higher than risk of suicide among male physicians.^1,2^ Somewhat consistent with this notion, other international meta-analytic evidence suggests female physicians experience higher suicide mortality than the female general population, while male physicians experience lower suicide mortality than the male general population.^3^ Regarding nurses, the single international systematic review on nurse suicide suggests nurses experience higher suicide mortality than certain occupational groups and the general population.^4^ Psychological autopsy studies of physician and nurse suicide may provide insights into contributing factors and circumstances that precede death.

The inception of psychological autopsy may be traced to the Los Angeles Suicide Prevention Center, where Edwin Shneidman developed the term “psychological autopsy” while working collaboratively with Robert Litman and Norman Farberow.^5^ More specifically, Theodore Curphey, the Los Angeles County Chief Medical Examiner-Coroner, consulted Shneidman, Litman and Farberow and requested assistance in determining mode of death among equivocal cases (i.e., deaths which were not clear about mode).^5^ To determine mode of death among equivocal cases (e.g., “accident” vs “suicide”), Shneidman, Litman and Farberow conducted retrospective analyses that examined psychological aspects of death, including decedents’ mindset and intention at time of death.^5^

Psychological autopsy represents a behavioral science that follows an objective process to facilitate impartial investigation into the psychological aspects of a death.^6^ This process includes integrating a variety of sources (e.g., interviews with survivors, police/coroner/medical records, legal documents, decedent’s personal documents) to identify and examine: (1) demographic information; (2) details on the death; (3) history (e.g., medical, mental health, suicide attempt(s), criminal); (4) family death history; (5) personality and lifestyle; (6) typical responses to stress and emotional challenges; (7) stress, pressures, or anticipated troubles within the preceding 12 months; (8) role of alcohol or drugs in life and death; (9) nature of interpersonal relationships; (10) thoughts or fears pertaining to death, accidents, or suicide; (11) changes in habits, behaviors, or routines before death; (12) successes or other plans; (13) intention (i.e., role of the decedent in their own death); (14) lethality; and (15) informants’ reactions to the death.^7^ The retrospective psychological investigation may provide insights into why an individual died, how the individual died, and the most accurate mode of death.^7,8^

Because both physicians and nurses experience disproportionate risk for suicide, the aim of this review was to identify and examine psychological autopsy studies of physician and nurse suicide, with the overriding goal of better understanding circumstantial and psychological aspects of death. To the best of our knowledge, this represents the first review to examine psychological autopsy studies of physicians and nurses. Taken together, this review is timely and relevant, as information derived from objective psychological investigation of physician and nurse suicide decedents may help to inform knowledge regarding circumstantial and psychological risk factors, which may ultimately inform prevention and intervention strategies.

## Methods

Following Shneidman’s^6^ definition, for the purposes of this paper, psychological autopsy was defined as a behavioral science that follows an objective process to examine psychological aspects of a death. A variety of search strategies were utilized to identify psychological autopsy studies on physician and nurse suicide. An exhaustive literature search was conducted using PubMed, PsycInfo, and CINAHL. A combination of keywords were used during the search of medical databases (i.e., *suicide*, *occupation*, *physician*, *doctor*, *nurse*, *registered nurse*, *nursing*, *healthcare provider*, *healthcare worker*, *healthcare professional*, *health practitioner*, *clinician*, *psychological autopsy*, *medicolegal*, *forensic*, *psychopathology*, *case control*).

During the literature search, reviews that examined suicide according to occupation, physician suicide, nurse suicide, and psychological autopsy were identified and separated. These reviews’ reference lists were hand-searched to identify relevant articles. The search for articles not included in any of these reviews continued, and each time a psychological autopsy study was identified, the references from the study were hand-searched to identify additional relevant articles. To supplement literature searches, researchers with expertise in psychological autopsy were identified, and these researchers’ academic websites and CVs were hand-searched to identify relevant articles. Similarly, suicide-related publications from national and international organizational websites were hand-searched to identify relevant articles.

All years and countries were searched without restriction. Articles in the English language were included. Psychological autopsy studies that contained physicians broadly were included (e.g., medical students, physicians). Psychological autopsy studies that contained nurses broadly were included (e.g., nursing students, registered nurses).

Studies that examined physician or nurse suicide using a psychological autopsy approach, which included formal interviews with survivor informants, were included. Studies were excluded if they did not describe the methods or data sources used to conduct the study. Psychological autopsy studies were excluded if they included physicians or nurses, but also included other individuals, and did not differentiate findings by occupation.

## Results

Eight psychological autopsy studies on physician suicide and one psychological autopsy study on nurse suicide were included. Physician suicide decedents from England and Wales, Finland, Thailand, and the United States were examined using psychological autopsy. Nurse suicide decedents from England and Wales were also examined using psychological autopsy. A narrative summary, which was organized according to occupation and country, presents important contextual information per article. This foundational narrative summary is then followed by an integrated narrative synthesis.

Sociodemographic characteristics (i.e., sex, age, race, ethnicity, marital status), occupational status at time of death (e.g., actively employed, retired), method(s) of suicide, location of suicide, and presence of a suicide note were inconsistently, and often infrequently, reported across studies (see supplementary file). Alternatively, histories, contributing factors, and circumstances preceding death were consistently reported across studies (see text).

### Physician Suicide in England and Wales

***Hawton et al.***^9^ (study period 1991-1993; female and male sample; *n*=38 decedents): Physician suicide decedents were examined using information from the Office for National Statistics, death certificates, coroner notes of evidence, inquest records, clinical notes from general practitioners, questionnaires from general practitioners, psychiatric case notes, and interviews with survivors (i.e., relatives, friends). Prior to suicide, 11 of 31 (35.5%) decedents stated they were considering suicide, and in 7 of 11 (63.6%) cases, these statements typically occurred within one week of death. Regarding decedents, 25 of 29 (86.2%) experienced a psychiatric disorder at time of death, with depressive disorders being most common. Co-morbidity of psychiatric disorders also occurred, and 5 of 29 (17.2%) decedents experienced concomitant psychiatric disorders, particularly affective and alcohol use disorders. Further, 8 of 29 (27.6%) decedents experienced primary or secondary diagnoses of alcohol and/or drug use disorder. In 3 of 16 cases (18.8%), decedents experienced personality disorder. Among decedents in contact with a general practitioner, 13 of 27 (48.1%) had seen this provider within three months of death, and in 8 of 13 (61.5%) of cases, the reason for the most recent visit included emotional or psychiatric concerns. Further, 13 of 25 (52%) decedents had consulted a psychiatrist in the 12 months preceding death. Regarding psychiatric treatment, 15 of 38 (39.5%) decedents were receiving some mode of treatment from a mental health specialist (nine cases) or their general practitioner (six cases) at time of death. Among the 21 decedents with depressive disorder, six had been receiving treatment from their general practitioner, eight had been receiving treatment at an outpatient clinic, and seven received no treatment. Among the 14 decedents who had been receiving treatment for their depressive disorder, most were prescribed antidepressant medication (12 cases), while two decedents self-prescribed antidepressant medication. Regarding mental health history among decedents, 20 of 29 (69%) had previous psychiatric history, which included psychiatric hospitalization (7 of 20 cases), and 9 of 28 (32.1%) had previous suicide attempts. In 25 of 35 (71.4%) cases, decedents experienced intersecting work-related problems prior to death, including feeling overwhelmed by the volume of work (eight cases), complaints (seven cases), long hours (six cases), and inability to cope with professional demands (six cases). Additional intersecting problems preceding death included relationship concerns in 14 of 35 (40%) cases, financial concerns in 10 of 35 (28.6%) cases, concerns regarding friends and family in 8 of 35 (22.9%) cases, physical health concerns in 4 of 35 (11.4%) cases, bereavement in 4 of 35 (11.4%) cases, legal concerns in 3 of 35 (8.6%) cases, and housing concerns in 3 of 35 (8.6%) cases. Taken together, intersecting problems were worsened by concomitant depressive disorder or alcohol or drug use disorder.

### Physician Suicide in Finland

***Lindeman et al.***^10^ (study period 1987-1988; female and male sample; *n*=7 decedents): Physician suicide decedents were examined using psychiatric records, medical records, social agency records, police reports, forensic reports, toxicology reports, suicide notes, and interviews with the decedents’ relatives and health care providers. Decedent one experienced physical health concerns and insomnia. Decedent one’s wife suspected her husband was depressed due to these health concerns and his inability to secure a desired job in another city. Similarly, decedent one’s supervisor suspected the underlying cause of suicide was severe depression. Decedent two experienced chronic physical illnesses, and three months prior to his suicide, he experienced a stroke that resulted in disabling hemiplegia. Decedent two’s wife stated that her husband believed individuals suffering from severe illness have the right to die, particularly when there is “nothing left but death” (p.137). Decedent three had no history of suicide attempt, somatic disease, or psychiatric disorder; however, four days before her suicide, she consulted a psychiatrist for concerns regarding depression, although she declined psychotherapy. Decedent three also avoided confiding in friends, family and colleagues, in part, as she experienced difficulties in personal and professional relationships. Decedent four had four previous suicide attempts and experienced depression. Decedent four received psychiatric hospitalization on one occasion, and psychotherapy was recommended, although she never entered psychotherapy. Decedent four’s husband stated that the reasons for his wife’s suicide included depression, rheumatoid arthritis that affected her work and hobbies, her uncle’s suicide, and her parents’ deaths. Decedent five experienced chronic cardiac issues that resulted in numerous emergency hospital admissions, including a second heart attack within one month of her suicide. Decedent five’s suicide note described her fear of dying due to chronic cardiac issues. Decedent six experienced rheumatoid arthritis and depression, although she never received formal medical treatment or psychotherapy, as her family members were also physicians and help was not sought from outside providers. Decedent sixes husband stated that his wife’s suicide was due to the death of her identical twin sister within the previous 12 months, which worsened her untreated depression. Decedent seven experienced numerous somatic diseases, depression, and had several previous suicide attempts. Decedent seven had two previous psychiatric hospitalizations, and after the second hospitalization, she entered private psychotherapy. In the year preceding suicide, decedent seven experienced cerebrovascular disease that resulted in medical hospitalization, physical disability, and severe emotional distress.

***Lindeman et al.***^11^ (study period 1987-1988; female and male sample; *n*=7 decedents): Using the same data sources, Lindeman et al.^11^ further examined mental disorders and mental disorder treatment among the seven physician suicide decedents described by Lindeman et al.^10^ Among the seven decedents, one died by suicide after killing her son with medications. Further, five experienced disabling physical health conditions. All decedents experienced a current mood disorder at time of death; specifically, four had depressive disorder, one had major depression, and two had bipolar disorder and were depressed at time of death. The two decedents with bipolar disorder had previous suicide attempts. At time of death, two decedents had pursued help for somatic symptoms as outpatients at general hospitals. Another decedent had pursued help for insomnia and somatic symptoms and was prescribed a low-dose antidepressant for insomnia. One decedent did not pursue help for depression and somatic symptoms. Only two decedents had pursued help for their current depression, although neither were prescribed antidepressants. Taken together, three decedents were prescribed psychiatric medication at time of death (i.e., Doxepin, Alprazolam and Oxazepam, Diazepam); however, no decedents had received sufficient treatment with antidepressant or mood-stabilizing medications. Further, only one decedent had received psychotherapy at time of death.

### Physician Suicide in Thailand

***Visanuyothin et al.***^12^ (suicides occurring before 2002; female and male sample; *n*=18 decedents): Physician suicide decedents were examined using records from the Centre for Continuing Medical Education, records from the Medical Council of Thailand, medical records, coroner reports, police reports, and newspaper reports. Interviews with decedents’ relatives, friends, or colleagues were also utilized. Decedents experienced overlapping circumstances preceding death; specifically, 12 experienced interpersonal conflict or loss, seven experienced work or legal problems, six experienced physical health concerns, five experienced mental health concerns, two experienced substance use disorder, and two experienced stress associated with professional demands. Interpersonal conflict or loss represented the specific circumstance that most strongly contributed to death in six cases, followed by mental health concerns in four cases, work or legal problems in three cases, physical health concerns in two cases, and stress associated with professional demands in one case. Regarding mental health concerns, six decedents had no diagnosed psychiatric disorder, four experienced a severe depressive episode without psychotic symptoms, two experienced adjustment disorders, two experienced mental and behavioral disorders due to use of sedatives or hypnotics, one experienced a depressive episode, one experienced schizophrenia, and one experienced organic delusional disorder.

### Physician Suicide in the United States

***DeSole et al.***^13^ (study period 1965-1968; for the psychological autopsy component of the study, data on sex of decedents was not reported; *n*=7 decedents): Physician suicide decedents from the Northeast were examined using data from the American Medical Directory, obituaries from the Journal of the American Medical Association, and interviews with decedents’ physician colleagues, other professional colleagues, relatives, and/or friends. Of the seven decedents, five were married, and three experienced problems in the marriage. No decedents experienced financial concerns or physical health concerns. Six decedents had no history of psychiatric disorder, although one decedent had history of previous suicide attempt. Current depressive symptoms were common among all seven decedents. One decedent experienced current concomitant alcohol use disorder and also used barbiturates. In six of the seven cases, survivors mentioned that signs of depression were most apparent in the two to five months preceding suicide. Three decedents were receiving psychiatric treatment at time of death, and one decedent had history of inpatient psychiatric hospitalization. The remaining three decedents were not believed to be receiving psychiatric treatment at time of death, although survivors mentioned they were not surprised when they learned the decedents’ died by suicide.

***Crawshaw et al.***^14^ (study period 1976-1977; male sample; *n*=6 decedents): Male physician suicide decedents from Oregon, who were on probation at time of death, were examined using data from the Oregon Board of Medical Examiners and interviews with the closest surviving relative. The psychiatrist of one decedent was also interviewed. None of the decedents were natives of Oregon, and most had practiced medicine in several areas of the U.S. Before probation, varying degrees of depression were present among all decedents, and all decedents were formally diagnosed with a psychiatric disorder. Older decedents experienced situational depression due to loss of a spouse or physical health. Younger decedents experienced emotional concerns and depression associated with instability during their early years. Most decedents were described as anxious, “introverted”, and “sensitive” (p.1916). Decedents did not respond well to stress, as stress often resulted in paranoia or manic-depressive behaviors. Among decedents, five experienced substance use disorder (i.e., amphetamines or meperidine hydrochloride) and four experienced alcohol use disorder. Four decedents were divorced at least one time, and most decedents had limited social, community, and religious connections. Several decedents were unable to achieve the professional goals they established for themselves, and they were often professionally isolated (e.g., colleagues ended cooperative practices, decedents were removed from positions). Across all decedents, there was no family history of suicide; however, four decedents had a history of previous suicide attempt.

***American Medical Association (AMA) Council on Scientific Affairs***^15^ (study period 1980-1981; female and male sample; *n*=15 decedents): Medical student and physician suicide decedents from five states were examined using the AMA’s Physician Records section, which includes all death reports, obituaries, and direct reports from these five states. Interviews with spouses, relatives, or other survivors were also utilized. There were no remarkable differences in use of alcohol or drugs between suicide decedents and physicians who died of natural causes (i.e., controls), although 26% of suicide decedents increased alcohol consumption at time of death, as compared to 8% of controls. Similarly, there were no substantial differences in propensity to overwork between suicide decedents and controls. Alternatively, 86% of suicide decedents experienced recent personal and professional losses, while no controls experienced losses. Further, 60% of suicide decedents experienced financial concerns, as compared to 22% of controls. While 33.3% of suicide decedents and 25% of controls experienced trouble in their practices (e.g., malpractice suit), all suicide decedents had their medical licenses revoked or hospital affiliations severed, while controls retained their licenses and affiliations. Among suicide decedents, 20% were viewed as “practicing incompetently” and 13% were viewed as “mildly irresponsible”, while no controls were viewed as “practicing incompetently” or “mildly irresponsible” (p.39). Regarding career satisfaction, 20% of suicide decedents and 52% of controls were “deeply satisfied”, while 33% of suicide decedents and no controls were “bitterly dissatisfied” (p.39). Only 33% of suicide decedents were viewed as cooperative with colleagues, as compared to 78% of controls. Regarding sources of satisfaction, 13% of suicide decedents and 30% of controls identified family, while 27% of suicide decedents and 3% of controls identified independent recreation. In 53% of cases survivors stated the suicide was “definitely foreseeable”, and in 33% of cases survivors stated the suicide was “possibly foreseeable” (p.39). Further, 60% of suicides were perceived as “possibly preventable” (p.39). Regarding clues preceding suicide, 50% of survivors reported changes in decedents’ mood, and 19% reported changes in decedents’ work routines.

***AMA Council on Scientific Affairs***^16^ (study period 1982-1984; female and male sample; *n*=142 decedents): Medical student and physician suicide decedents were examined using the AMA’s Physician Records section, which includes all death reports, obituaries, and direct reports. Interviews with family members, friends, colleagues, or other survivors were also utilized. Most survivors (84%) believed there were clues preceding suicide. Nearly half of decedents stated they were contemplating suicide in the two years preceding death, most frequently to their spouse (62%) or a friend (32%). Survivors (37%) also reported changes in decedents’ mood preceding death. However, 16% of survivors reported that there were no clues. The most prevalent reasons for suicide included escaping mental pain (94%), physical pain (26%), or to benefit another (23%), with reasons overlapping in several cases. Suicide decedents had more challenging patients when compared to physicians who died of other causes (i.e., controls). Among suicide decedents, 77% experienced chronic mental or physical health concerns, as compared to 65% of controls. Regarding alcohol and drug use, 34% of suicide decedents experienced problematic use, as compared to 14% of controls. Suicide decedents encountered more social problems stemming from problematic alcohol use, as compared to controls. Suicide decedents were also more likely than controls to drink until unconscious. Among suicide decedents, 56% self-prescribed a psychoactive drug, as compared to 22% of controls. Suicide decedents were more likely than controls to be critical of themselves and blame themselves for personal illnesses. Among suicide decedents, 42% had consulted a mental health provider at time of death, as compared to 7% of controls. Among suicide decedents, 33% experienced historic psychiatric hospitalization once and 58% were hospitalized more than once, as compared to 7% and 0% of controls, respectively. Regarding historic suicide attempts, 34% of suicide decedents made previous attempts, as compared to 6% of controls. Suicide decedents were more likely than controls to have mentioned they were considering suicide and be less optimistic about the future. Suicide decedents were also more likely than controls to have experienced professional and financial losses in the two years preceding death. Suicide decedents had fewer friends than controls, and they received less emotional support from children, friends, colleagues, and parents. However, when compared to controls, suicide decedents provided less emotional support to their spouse, children, friends, and colleagues. When compared to controls, suicide decedents were more likely to physically assault their spouse. Among suicide decedents, 14% experienced emotional concerns before age 18, as compared to 4% of controls. When compared to controls’ families, alcohol use disorder and mental health disorders were more common among suicide decedents’ families.

### Nurse Suicide in England and Wales

***Hawton et al.***^17^ (study period 1994-1997; female sample; *n*=106 decedents): Female nurse suicide decedents were examined using information from the Office for National Statistics, death certificates, coroner notes of evidence, inquest and pathology reports, clinical notes from general practitioners, questionnaires from general practitioners, psychiatric case notes, and interviews with survivors (i.e., spouse or partner, parent(s), sibling, friend, daughter, psychiatrist). Interviews were also conducted with living control nurses, matched for age, specialty, and seniority. Among decedents, 69 of 97 (71.1%) had history of contact with psychiatric services, and 52 of 97 (53.6%) had history of psychiatric hospitalization. At time of death, 46 of 101 (45.5%) decedents were in current contact with psychiatric services. Further, 63 of 99 (63.6%) decedents had history of deliberate self-harm, and among those who self-harmed, 38 of 60 (63.3%) self-harmed numerous times. Among decedents in contact with a general practitioner, reasons for the most recent visit included emotional concerns in 52 of 95 cases (54.7%), physical health concerns in 37 of 95 cases (38.9%), or both emotional and physical health concerns in 6 of 95 cases (6.3%). At time of death, 61 of 99 (61.6%) decedents had prescriptions for psychotropics. Among decedents, 27 of 101 (26.7%) had physical health concerns at time of death (e.g., pain, asthma, epilepsy). In 38 of 42 cases (90.5%), informants suggested decedents experienced a current psychiatric disorder at time of death, as compared to 6 of 84 (7.1%) of controls. The leading diagnosis among nurse suicide decedents was affective disorders (i.e., depressive episode), followed by alcohol use disorders. Co-morbidity of current psychiatric disorders was present in 12 of 42 cases (28.6%), most frequently, concomitant affective and alcohol use disorders (seven cases). Further, in 16 of 42 cases (38.1%), informants suggested decedents experienced a current personality disorder at time of death, as compared to 1 of 84 (1.2%) controls. Among the 16 nurse suicide decedents with personality disorder, concomitant psychiatric disorder was present in all 16 cases. Taken together, when compared to living control nurses, nurse suicide decedents were more likely to have experienced current psychiatric disorder, current personality disorder, previous deliberate self-harm, previous psychiatric disorder, co-morbidity of psychiatric disorders, and co-morbidity of psychiatric and personality disorders. Additionally, when compared to living control nurses, nurse suicide decedents were more likely to have worked as a nurse for 1-5 years, been unmarried, have no children, not lived in one’s own home, lived alone, have moved two or more times in the previous five years, have no confidant, been a smoker at time of death, consumed more than 50 units of alcohol per week, and experienced financial problems in the previous 12 months. While there were modestly more work problems among living control nurses as compared to nurse suicide decedents (82.1% vs 66.7%), nurse suicide decedents experienced work overload (28.6%), clashes with work colleagues and/or supervisors (21.4%), limited occupational support (19.1%), and excessive work responsibilities (16.7%) in the previous 12 months.

### Narrative Synthesis

#### History of mental health concerns

Eight psychological autopsy studies on physician suicide and one psychological autopsy study on nurse suicide were examined. While physician and nurse suicide decedents from different global regions were examined, and while there were differences with respect to study periods, findings were somewhat consistent across studies. Regarding mental health history, physician suicide decedents often had previous psychiatric history, which included psychiatric hospitalization ([33% hospitalized]^16^; [14.3% history, 14.3% hospitalized]^13^; [69% history, 35% hospitalized]^9^; [28.6% history, 28.6% hospitalized]^10^). Similarly, nurse suicide decedents frequently had previous psychiatric history, which included psychiatric hospitalization ([71.1% history, 53.6% hospitalized]).^17^ Further, physician suicide decedents often had history of suicide attempt ([34%]^16^; [66.7%]^14^; [14.3%]^13^; [32.1%]^9^; [28.6%]^10^; [28.6%]^11^). Nurse suicide decedents also frequently had history of deliberate self-harm ([63.6%]).^17^

#### Mental health concerns at time of suicide

With respect to current mental health, most physician suicide decedents experienced current psychiatric disorder at time of death ([100%]^14^; [100%]^13^; [86.2%]^9^; [100%]^10^; [100%]^11^; [66.7%]^12^). Most nurse suicide decedents also experienced current psychiatric disorder at time of death ([90.5%]).^17^ Among physician suicide decedents, depressive disorders were most common.^9–14^ Similarly, among nurse suicide decedents, a depressive episode was most common.^17^ Physician and nurse suicide decedents sometimes experienced concomitant psychiatric disorders, particularly affective and alcohol use disorders ([66.7%]^14^; [17.2%]^9^; [28.6%]^17^). More broadly, primary or secondary diagnoses or issues related to alcohol and/or drug use disorder were mentioned across several physician and nurse studies ([34%]^16^; [83.3%]^14^; [14.3%]^13^; [27.6%]^9^; [21.4%]^17^; [11.1%]^12^). Alcohol consumption among physician suicide decedents was also reported to have increased at time of death ([26%]).^15^

#### Contact with healthcare providers

Regarding healthcare contacts, among physicians and nurses in contact with a general practitioner, the central reason for the most recent visit included emotional concerns ([61.5%]^9^; [54.7%]^17^). Some physician suicide decedents had consulted a mental health specialist at time of death ([42%]^16^; [42.9%]^13^; [52%]^9^; [28.6%]^11^). More specifically, with respect to mental health treatment, some physician suicide decedents were receiving a form of treatment at time of death ([42.9%]^13^; [39.5%]^9^). One study did not specify treatment(s);^13^ however, in another study, among physicians being treated for depressive disorder, most had been prescribed an antidepressant, and two decedents self-prescribed an antidepressant.^9^Self-prescribing^16^ and physician family member prescribing^11^ were mentioned in other physician studies as well. Alternatively, another study reported that only three physician suicide decedents had been prescribed psychiatric medication at time of death, and no decedents had received sufficient treatment with antidepressant or mood-stabilizing medications.^11^ Regarding mental health treatment among nurses, several nurse suicide decedents were in contact with and were receiving care from psychiatric services at time of death ([45.5%]).^17^ Nurses frequently had prescriptions for psychotropics at time of death ([61.6%]).^17^ More specifically, nurses had been prescribed antidepressants, minor tranquilizers or hypnotics, and major tranquilizers.^17^

#### Circumstances preceding suicide

Regarding circumstances preceding physician suicide, physician suicide decedents experienced: (1) physical health concerns^9–12,14,16^; (2) mental health concerns^9,12–14,16^; (3) work-related problems or losses^9,12,14–16^; (4) concerns regarding alcohol or substances^9,12,14,16^; (5) relationship concerns or losses^9,12–14^; (6) financial concerns or losses^9,15,16^; (7) concerns regarding friends and family^9,16^; (8) legal concerns^9,12^; (9) housing concerns^9^; (10) bereavement^9^; and (11) personal losses.^15^ Regarding related circumstances preceding nurse suicide, nurse suicide decedents were more likely than controls to: (1) experience mental health concerns; (2) consume more than 50 units of alcohol weekly; (3) experience financial concerns; (4) have no confidant; (5) live alone; (6) move two or more times in the previous five years; and (7) work for fewer years as a nurse.^17^

With respect to specific work-related circumstances in particular, physician suicide decedents: had challenging patients^16^; were unable to secure a desired job or accomplish a desired professional goal^10,14^; worked long hours^9,10^; were overwhelmed by the volume of work^9^; experienced difficulties in professional relationships^10,14^; received limited support from colleagues^16^; were unable to cope with professional demands^9^; practiced incompetently or irresponsibly^15^; experienced complaints or malpractice suits^9,15^; were on probation, had their medical license revoked, or had their hospital affiliation severed^14,15^; and were dissatisfied with their career^15^. Regarding specific work-related circumstances among nurses, nurse suicide decedents were early in their nursing career (i.e., 1-5 years), had disputes with work colleagues and/or supervisors, received limited occupational support, had excessive work responsibilities, and experienced work overload.^17^

#### Disclosing intent to die by suicide and other situational circumstances

With respect to more direct clues preceding suicide, survivors mentioned that physician suicide decedents stated that they were contemplating suicide ([nearly 50%]^16^; [35.5%]^9^). Some survivors reported these statements were made one week before death,^9^ while other survivors reported these statements were made in the two years preceding death.^16^ Survivors also mentioned that there were changes in physician suicide decedents’ mood^15,16^ and work routines^15^ preceding death. Further, survivors mentioned that signs of depression among physician suicide decedents were most apparent in the two to five months preceding suicide.^13^ When viewed collectively, survivors believed that physician suicides were “definitely foreseeable” or “possibly foreseeable” and “possibly preventable” (p.39).^15^

## Discussion

Additional research on physician suicide is important, as international meta-analytic evidence largely suggests female and male physicians experience higher suicide mortality than the female and male general population, respectively.^1,2^ Psychological autopsy study findings on physician suicide decedents are largely congruent with Silverman’s^18^ profile of a physician with elevated risk for suicide. Specifically, Silverman^18^ suggests historic or current mental health concerns, depression, alcohol or substance misuse, physical health concerns, and work-related problems elevate risk of suicide among physicians. Additional research on these overlapping findings between psychological autopsy studies and Silverman’s^18^ profile of physicians at risk for suicide may be important. To enhance cross-study comparison regarding overlapping findings, it may also be important to increasingly utilize standardized definitions of mental health diagnoses, alcohol and/or substance misuse, and circumstances preceding suicide; in part, as mental health concerns and life events have been inconsistently defined and assessed across the broader psychological autopsy literature.^19^ Further, based on recommendations derived from the literature more broadly, it may also be important to utilize a systematic process regarding timing of survivor informant interviews, selection of informants, interview content, and training of interviewers.^19–22^

Additional research may also be important in updating the evidence base, as the most recent psychological autopsy study on physician suicide decedents was conducted 22 years ago (i.e., Visanuyothin et al.^12^). Updating the evidence base may be important, as physicians have encountered unprecedented challenges in recent years, particularly with respect to the global pandemic, and it is important to understand how recent occupational difficulties might impact risk for suicide. Further, updating evidence may be important, as the World Health Organization^23^ suggests there is a health worker shortage globally that is expected to increase, and demands on physicians globally are high and may continue to increase. Updated psychological autopsy study evidence may provide insights into the potential impact of recent and future global events on multifaceted risk factors for suicide among physicians. Collectively, six psychological autopsy studies mentioned work-related factors,^9,10,12,14–16^ although in most cases, these descriptions were quite broad and there were very few examples of specific work-related circumstances that might contribute to suicide. Broadly, evidence suggests that healthcare workers are frequently exposed to difficult and stressful working conditions (e.g., long hours, on-call work, unpredictable work environments, administrative burdens, increased patient loads, mounting pressures from patients and employers), which may result in poor physical- and mental-health,^24^ although evidence is quite scoping, and additional research regarding specific occupational factors that most significantly impact risk for suicide among physicians is important. Taken together, psychological autopsy studies provide a strong framework for better understanding work-related circumstances that precede suicide, and this represents a timely and important research need, given recent global events and present research gaps.

Further, sample sizes in physician studies ranged from six^14^ to 142^16^ and additional psychological investigations with large samples may be important in building the evidence base. Somewhat consistent with this notion, a research consensus statement from the American Foundation for Suicide Prevention (AFSP) recommended conducting a large psychological autopsy investigation of physician suicides to further explore risk and protective factors, help seeking behaviors, adequacy of treatment, and treatment adherence.^25^ Following AFSP recommendations, it may be important to focus specifically on help seeking, adequacy of treatment, and treatment adherence; in part, as collective psychological autopsy evidence largely suggests physician suicide decedents experienced mental health concerns that were undertreated or untreated. Further, psychological autopsy evidence additionally suggests physical health concerns were prevalent. Psychological autopsy studies may benefit from additional augmentation with electronic health record data, which may increasingly permit examination of healthcare utilization more proximally. Finally, following AFSP recommendations, it may also be important to concurrently focus on identifying protective factors, as most psychological autopsy studies on physician suicide focused nearly exclusively on risk factors.

Additional investigations of physicians from different global regions may also be important in determining generalizability of findings; in part, as international meta-analytic evidence suggests risk of suicide among physicians is not homogeneous across countries, with risk being highest in the U.S. when compared to 19 other countries.^1^ Across the literature more broadly, historic psychological autopsy studies often have not fully considered sociodemographic factors and the sociocultural environment,^19^ and it may be prudent to intentionally explore these factors while examining physician suicide decedents from additional countries. Collectively, additional research is important, as further examining circumstances and psychological factors that precede physician death by suicide are important in informing interventions designed to prevent physician suicide.

When compared to living control nurses, nurse suicide decedents experienced more historic and current psychiatric disorder, personality disorder, previous suicide attempt, alcohol use, and financial concerns.^17^ Psychological autopsy findings are congruent with review evidence. Specifically, an international integrative review suggests mental health concerns, alcohol and substance misuse, and financial concerns represent correlates of suicidal ideation among nurses.^26^ Further, this global review suggests financial concerns, personality disorder, depressive symptoms, and previous suicide attempt represent correlates of suicide attempt among nurses.^26^ Finally, this international review suggests mental health history, current mental health concerns, and alcohol misuse represent correlates of death by suicide among nurses.^26^ Additional research on these overlapping circumstances may be important; in part, as financial concerns (i.e., correlate of suicidal ideation and suicide attempt), alcohol misuse (i.e., correlate of suicidal ideation and death by suicide), and mental health concerns (i.e., correlate of suicidal ideation, suicide attempt, and death by suicide) intersected across several levels.

Additional research on these overlapping circumstances may also be important, because despite exhaustive searches, only one psychological autopsy study on nurse suicide was identified. Limited availability of data on nurse suicide reflects a concern across the global literature. Specifically, the single systematic review on suicide among nurses included all empirical research designs, and included associated factors, suicide risk, interventions, suicidal ideation, suicide attempt and death by suicide, although only 100 total global studies were identified from 1996 to 2021.^4^ Because this single systematic review suggests nurses experience higher suicide mortality than certain occupational groups and the general population,^4^ additional research is important in expanding the evidence base and providing further insights into nurse suicide globally.

The single psychological autopsy study on nurse suicide decedents included female nurses from England and Wales,^17^ and additional research may be important in updating the evidence base, as this study was conducted 27 years ago (i.e., Hawton et al.^17^). Like physicians, nurses have encountered unprecedented challenges in recent years, particularly with respect to the global pandemic, as nurses were on the front lines providing direct patient care. The World Health Organization^27^ confirms that nurses are essential in delivering patient care, and nurses are often the only health worker that patients encounter in many settings globally. More broadly, evidence suggests that healthcare workers are often exposed to high-stress, demanding working conditions (e.g., shift work, double shifts, risk for exposure to chemicals and pathogens, risk of injury due to patient handling, exposure to patient suffering and death), which may lead to declines in physical- and mental-health,^24^ although evidence is quite broad, and additional research regarding specific occupational factors that most significantly impact risk for suicide among nurses is important. Taken together, updated psychological autopsy study evidence may provide insights into the potential impact of increasing demands on nurses and recent global events on multifaceted risk factors for suicide among nurses. Because psychological autopsy studies provide a solid framework for better understanding work-related circumstances that may precede and/or contribute to suicide, this represents an important research need, given recent global events and current research gaps.

Further, additional psychological investigations that include both female and male nurse suicide decedents from different global regions may be also important in building the evidence base and in determining generalizability of findings. The single psychological autopsy study of nurses included female nurse suicide decedents only, and concurrent inclusion of male nurse suicide decedents is important in building the evidence base. Further, because the World Health Organization^28^ confirms that distribution of nurses and scope of practice among nurses varies fairly considerably globally, additional research will be important in identifying if circumstantial and psychological suicide risk and protective factors among nurses vary by region. Collectively, additional research is important, as more firmly identifying circumstances and psychological factors that precede female and male nurse death by suicide are important in informing interventions designed to prevent nurse suicide.

## Conclusion

Physician and nurse suicide decedents often had history of mental health concerns, psychiatric hospitalization, and suicide attempt(s). Physician and nurse suicide decedents also often experienced current mental health concerns at time of death, particularly depressive disorders. Alcohol and substance use disorder sometimes occurred concomitantly. While some physician and nurse suicide decedents had consulted a general practitioner or mental health specialist, across these particular studies, less than half had been receiving treatment at time of death. Further, given important evidence gaps, it is difficult ascertaining broader adequacy of mental health treatment. Physician suicide decedents frequently experienced physical health concerns, mental health concerns, work-related problems, concerns regarding alcohol or substances, and relationship concerns preceding death. Regarding circumstances preceding nurse suicide, nurse suicide decedents were more likely than controls to experience mental health and financial concerns, consume more than 50 units of alcohol weekly, be isolated, and relocate frequently. Taken together, psychological autopsy studies of physician and nurse suicide may provide important implications for research, surveillance, prevention, and intervention.

## Supplemental material

Supplemental material - Psychological autopsy studies of physician and nurse suicide: A narrative summary and synthesis of circumstantial and psychological aspects of death Supplemental material for Psychological autopsy studies of physician and nurse suicide: A narrative summary and synthesis of circumstantial and psychological aspects of death by Elizabeth Kreuze, Elizabeth I. Merwin, Maureen Underwood and Janet York in Journal of Public Health Research.
