# Supplementary material for: Psychological autopsy studies of physician and nurse suicide: A narrative summary and synthesis of circumstantial and psychological aspects of death
Source: J Public Health Res. 2026 Jul 3;15(3):22799036261465285. doi: 10.1177/22799036261465285 (PMC13332251; doi:10.1177/22799036261465285)
Supplement: Supplemental material - Psychological autopsy studies of physician and nurse suicide: A narrative summary and synthesis of circumstantial and psychological aspects of death [file sj-pdf-1-phj-10.1177_22799036261465285.pdf]

Supplementary File: Articles, Sociodemographic Characteristics, and Related Variables

<!--Col Count:12-->Country and Article	Study Period	Occupation	Suicide Decedents	Sex	Age at Time of Death	Race and Ethnicity	Marital Status at Time of Death	Occupational Status at Time of Death	Method(s) of Suicide	Location of Suicide	Suicide Note
**England and Wales**											
Hawton et al., 2004	1991-1993	Physicians	*n*=38	*n*=10 females, *n*=28 males	*n*=14 were under 35 years (ranged from 23 to 71 years)	NR	*n*=18 married, *n*=11 single, *n*=4 divorced or separated, *n*=5 unknown	*n*=32 working, *n*=1 recently retired, *n*=5 potential of returning to medicine after a temporary leave (in two cases, leave was due to mental health concerns)	*n*=27 self-poisoning, *n*=10 self-injury, *n*=1 both self-poisoning and self-injury	NR	*n*=21 left a suicide note
Hawton et al., 2002	1994-1997	Nurses	*n*=106	*n*=106 female nurse suicide decedents	*n*=42 were 20-34 years, *n*=51 were 35-49 years, *n*=13 were 50-59 years	NR	*n*=39 single, *n*=35 divorced or separated, *n*=30 married, *n*=2 widowed	If the death certificate noted the decedent was retired, interviews with survivors were not conducted	*n*=72 self-poisoning, *n*=32 self-injury (e.g., hanging), and *n*=2 both self-poisoning and self-injury	NR	NR
**Finland**											
Lindeman et al., 1998	1987-1988	Physicians	*n*=7	*n*=5 females, *n*=2 males	*n*=2 were under 40 years, *n*=1 was under 50 years, *n*=1 was over 60 years, *n*=1 was aged 65 years, *n*=2 were over 70 years	NR	*n*=5 married; *n*=1 divorced and then re-married, but this spouse died a few months into the marriage; *n*=1 widowed	Prior to suicide, *n*=2 used medical leave (in one case due to rheumatoid arthritis, and in another case due to cardiac problems); *n*=1 received full disability pension due to depression	NR	NR	*n*=1 left a suicide note
Lindeman et al., 1999	1987-1988	Physicians	*n*=7	*n*=5 females, *n*=2 males	Mean age 56.9 years (ranged from 33 to 77 years)	NR	*n*=5 married, *n*=2 widowed	*n*=3 were actively practicing medicine, *n*=2 were retired, and *n*=2 did not have a medical license in Finland (not due to malpractice)	*n*=2 hanging, *n*=1 hanging under the influence of alcohol, *n*=1 firearm, *n*=1 barbiturate and digoxin overdose, *n*=1 barbiturate overdose, *n*=1 metoprolol overdose	NR	NR
**Thailand**											
Visanuyothin	Suicides	Physicians	*n*=18	*n*=1 female,	Mean age 41.4	NR	*n*=9 married, *n*=9	*n*=7 were working as a	*n*=6 hanging, *n*=5	NR	NR

<!--Col Count:12-->Country and Article	Study Period	Occupation	Suicide Decedents	Sex	Age at Time of Death	Race and Ethnicity	Marital Status at Time of Death	Occupational Status at Time of Death	Method(s) of Suicide	Location of Suicide	Suicide Note
et al., 2004	prior to 2002			*n*=17 males	years (ranged from 24 to 67 years)		single	general practitioner, *n*=11 were working as specialists	firearm, *n*=5 poisoning with IV injection, *n*=1 poisoning with overdose, *n*=1 jumping from a building		
**United States**											
AMA Council on Scientific Affairs, 1986	1980-1981	Physicians	*n*=15	Unclear (female and male sample)	NR	NR	NR	NR	NR	NR	NR
AMA Council on Scientific Affairs, 1987	1982-1984	Physicians	*n*=142	*n*=13 females, *n*=129 males	Mean age 49.3 years (ranged from 25 to 93 years)	NR	69% married, 22% separated or divorced	69% actively practiced medicine	46% firearm, 29% drug overdose, 11% hanging, 14% other means	62% at home, 11% hotel/motel room, 6% automobile	42% left a suicide note
Crawshaw et al., 1980	1976-1977	Physicians	*n*=6	*n*=6 males	*n*=4 were 35–44-year-olds, *n*=2 were 65-74-year-olds	NR	*n*=4 divorced	*n*=6 were on professional probation status	NR	NR	NR
DeSole et al., 1969	1965-1968	Physicians	*n*=7	NR	Mean age 39 years (ranged from 26 to 57 years)	NR	*n*=5 married	NR	NR	NR	NR

*NR=Not Reported

==========

PSYCHOLOGICAL AUTOPSY STUDIES OF PHYSICIAN AND NURSE SUICIDE
